# Notes and News

**Published:** 1898-08-13

**Authors:** 


### Notes and News.

(Collected and Communicated.)

Sir John Dickson Poynder, Bart., M.P., has been elected chairman of the committee of management of the Great Northern Central Hospital, in succession to the late Mr. C. T. Murdoch, M.P.

Mr. Licatza gave a matinée last week in aid of the Prince of Wales's Hospital Fund. Much interest has been excited by the contributions of the Princesses Margaret and Victoria of Connaught, who have each made a gift of £1 from their pocket money.

The foundation-stone has been laid of additional accommodation at the Margate Cottage Hospital. This has been possible through the Jubilee fund collected locally. The improvements to be made will place Margate in possession of a much more convenient and complete hospital.

We learn that the new 1898 stamps of the Prince of Wales's Hospital Fund will be ready on September 20th. Orders can now be received by all booksellers and chemists. The stamps, which bear a face value of 1s., 2s. 6d., 5s., and 10s. respectively, are each of different design and colour, especially selected by the Prince of Wales himself.

The gift of a site for a cottage hospital has been accepted by the people of Penrith, and the Diamond Jubilee Commemoration Fund having reached £1,515, it has been decided to commence the erection of the building, estimated to cost £1,412. Annual subscriptions have already been promised, and it is hoped that before the hospital is completed money to furnish it will be forthcoming.

The appointment of Mr. Thomas Morison Legge to fill the new post of Medical Inspector of Factories and Workshops is a very suitable one. Mr. Legge took honours in physiology at Oxford, and later studied medicine at Oxford, St. Bartholomew's Hospital, and at Vienna. He took the degree of Doctor of Medicine in 1894, and possesses exceptional knowledge of continental sanitation, and was secretary to the Royal Commission on Tuberculosis.

The Dieppe Infirmary has just been the scene of an alarming conflagration. The fire broke out in the wing where 150 aged patients were housed. The building was entirely destroyed, and another part of the infirmary injured. The patients were successfully removed by the hospital staff.

It is reported that the American army at Santiago is so weakened and shattered with malarial fever that an immediate move northwards is necessary if a most serious mortality is to be escaped. General Shafter's report gave 3,697 now on the sick list, the majority being sufferers from fever. There have been eight deaths from yellow fever.

Mr. Chaplin has introduced a timely Bill to the House of Commons making provision against the adulteration of "perishable" foods, such as milk and butter imported from abroad, as well as new and more stringent provisions with regard to samples taken by officers of the Board of Agriculture for analytical purposes. The Bill is aimed against the notorious additions and adulterations existent in foreign dairy products.

A terrible story comes from Vienna of the brutal treatment received by a supposed leper in Bohemia. The report runs that a man who was suffering from a rash mistaken for leprosy, was put in a pit by his fellow villagers, who then tried to burn him alive. He effected an escape, in spite of his injuries, only to be rediscovered and then thrown into the snow. Both his feet becoming frostbitten, he cut them off with his clasp knife. Finally he was found by a priest, and sent to a hospital at Vienna, where he now lies.

At the intermediate examination in medicine in July at the University of London the following gentlemen passed the examination for honours: In anatomy—First class, Thomas Copeland Savage (exhibition and a gold medal) (University College); second class, Sidney Herbert Bown (University College); third class, Walter Fedde Fedden (St. George's Hospital), Alec Leslie Matthews (London Hospital), and Alfred Bertram Soltau (London Hospital). Physiology and histology—First class, Arthur Edmunds, B.Sc. (exhibition and a gold medal) (King's College), and William Henry Wynn, B.Sc. (gold medal) (Mason University College); second class, Julius Meyer Bernstein (Owens College), Arthur Eastwood (Cambridge University and Owens College), and Alfred Bertram Soltau (London Hospital); third class, William Osborne Greenwood (Yorkshire College) and Stanley Hodgson (Guy's Hospital). Organic chemistry—Second class, Howard Welles Reynolds (University College). Materia medica and pharmaceutical chemistry—First class, Arthur Eastwood (exhibition and gold medal) (Cambridge University and Owens College), Arthur Edmunds (King's College), and Oskar Cameron Gruner (Owens College); second class, James Frederick Edmund Bridger (St. Mary's Hospital), Arthur Falconer Hayden (St. Mary's Hospital), William Payne (Westminster Hospital), and Alfred Bertram Soltau (London Hospital); third class, Thomas Henry Bourne Dobson (Owens College) and Friedrich Gröne (St. Bartholomew's Hospital).

